# An Integrated Platform Combining Immersive Virtual Reality and Physiological Sensors for Systematic and Individualized Assessment of Stress Response (bWell): Design and Implementation Study

**DOI:** 10.2196/64492

**Published:** 2025-03-04

**Authors:** Budhachandra Khundrakpam, Melanie Segado, Jesse Pazdera, Vincent Gagnon Shaigetz, Joshua A Granek, Nusrat Choudhury

**Affiliations:** 1 National Research Council Canada Boucherville, QC Canada; 2 Defence Research and Development Canada Toronto, ON Canada

**Keywords:** virtual reality, stress, physiological response, NASA-Task Load Index, cognitive demand, physical demand, vagal tone, heart rate variability

## Abstract

**Background:**

Stress is a pervasive issue in modern society, manifesting in various forms such as emotional, physical, and work-related stress, each with distinct impacts on individuals and society. Traditional stress studies often rely on psychological, performance, or social tests; however, recently, immersive virtual reality (VR), which provides a sense of presence and natural interaction, offers the opportunity to simulate real-world tasks and stressors in controlled environments. Despite its potential, the use of VR to investigate the multifaceted manifestations of stress has not been thoroughly explored.

**Objective:**

This study aimed to explore the feasibility of using a VR-based platform, *bWell*, to elicit multifaceted stress responses and measure the resulting behavioral and physiological changes. Specifically, we aimed to design various VR stress exercises based on neurocardiac models to systematically test cardiac functioning within specific contexts of self-regulation (executive functioning, physical efforts, and emotional regulation).

**Methods:**

The development process adhered to guidelines for VR clinical trials and complex health interventions, encompassing 3 phases: preparation, development, and verification. The preparation phase involved a comprehensive literature review to establish links between stress, the heart, and the brain, leading to the formulation of a conceptual model based on the Neurovisceral Integration Model (NVIM) and Vagal Tank Theory (VTT). The development phase involved designing VR exercises targeting specific stressors and integrating physiological sensors such as photoplethysmography (PPG) and electromyography (EMG) to capture heart rate variability (HRV) and facial expressions. The verification phase, conducted with a small number of trials, aimed to design a study and implement a workflow for testing the feasibility, acceptability, and tolerability of the VR exercises. In addition, the potential for capturing physiological measures along with subjective ratings of stress for specific dimensions was assessed.

**Results:**

Verification trials demonstrated that the VR exercises were well tolerated, with negligible cybersickness and high user engagement. The different VR exercises successfully elicited the intended stress demands, along with the physiological responses.

**Conclusions:**

The study presents a novel VR-based experimental setup that allows a systematic and individualized assessment of stress responses, paving the way for future research to identify features that confer stress resilience and help individuals manage stress effectively. While our conceptual model highlights the role of HRV in providing valuable insights into stress responses, future research will involve multivariate and machine learning analyses to predict individual stress responses based on comprehensive sensor data, including EMG and the VR-based behavioral data, ultimately guiding personalized stress management interventions.

## Introduction

### Background

A rising problem in our society is *stress*, with serious burdens imposed on individuals and society by different types of stressors, such as emotional stress, physical stress, and work stress [1,2]. The multifaceted nature of stress makes it even more problematic, with different stressors yielding different responses and consequences. For example, increased workload and time pressure has led to increased mental stress [3]. Similarly, in recent years, there has been an increase in emotional, behavioral, and psychological stress due to the COVID-19 pandemic [4,5].

Cumulative evidence from studies of stress neurobiology indicates individual variability in how people respond to stress. While for some individuals, stress can lead to negative outcomes (eg, deterioration of physical and mental health), other individuals may not be affected to that extent [6,7]. Identifying the risk factors that make a person vulnerable to negative consequences of stress would allow timely detection and intervention. By contrast, identifying and delineating the individual features that potentially confer stress resilience (defined as the ability of most people to maintain normal functioning even under extreme stress) is important in helping individuals reach their full potential. Factors that confer resilience include genetic predisposition, age, sex, prenatal environment, sleep, and exercise [8]. In terms of biological mechanisms, stress resilience has been attributed to a coordinated activity of the autonomic nervous system (ANS); the hypothalamic-pituitary-adrenal axis; the immune system; and the brain regions that control their activity, including the hypothalamus, amygdala, hippocampus, medial prefrontal cortex, and ventral tegmental area [9-11]. In particular, stress-induced activation of ANS has been linked to changes in heart rate and vasoconstriction [9]. In addition, stress responses via activation of sympathetic-adrenomedullary and hypothalamic-pituitary-adrenal axes have been observed in response to psychosocial and physical demands during intense exercise [12].

Given the complex coordinated involvement of several bodily systems (both central and peripheral), studies on stress resilience require measurement of attributes that can reflect these interactions. One such attribute is heart rate variability (HRV), which captures heart-brain interactions and ANS dynamics [13]. HRV is defined as the variation in the time intervals between consecutive heartbeats and is an emergent property of complex interactions among several interdependent physiological systems. Several studies have shown the usefulness of HRV in the analysis of stress that the body experiences during physical training and to increase insight into physiological recovery after training [14,15]. Measurement of HRV (using electrocardiogram [ECG] or photoplethysmography [PPG]) is an easy noninvasive technique, and therefore, it has been used to investigate stress-related changes in cardiovascular health in different conditions, including exercise [16] and sleep [17]. More importantly, studies on individuals with stress-related disorders (such as anxiety and depression) revealed altered HRV metrics, indicating impairment of neurocardiac function mediated by changes to the ANS [18]. In addition, integrating HRV with other modalities such as electroencephalography have shown the impact of stress on cardiac and brain health [19,20].

Traditionally, the impact of stress on biological measures such as HRV and brain activity is investigated after inducing a specific stressor (mainly in live scenarios) using psychological, performance, or social tests [21,22]. However, in recent years, the advancement of virtual reality (VR) and head-mounted displays has allowed the investigation of different stressors that are otherwise difficult to reproduce in real life. Using head-mounted displays, immersive VR offers a sense of presence and a more natural interaction, allowing multisensorial (visual, audio, and touch) simulation of real-world tasks with tight experimental control, enabling the study of specific stressors. In a study, VR was used to elicit stress in high-altitude simulations, and stress-related responses in the heart and brain were observed using ECG and electroencephalography, respectively [23]. In a VR study using ECG signals, a multilevel stress assessment (3 levels of stress) was performed using spectrograms and 1D ECG signals that performed better than traditional machine learning (ML) models [24]. In another example, playing 3D games (a combination of the first-person shooter and horror genres) in immersive VR was shown as an effective stressor for practicing stress management skills [25]. In alignment with these findings, a meta-analysis of 52 studies revealed that stress tasks in VR elicit a variety of physiological stress reactivity, highlighting the scope of VR in the study of stress [26].

### Objectives

Despite the advantages offered by VR (multisensorial—visual, audio, and touch simulation of real-world tasks), previous studies have mostly used VR to induce particular stressor at a time, for example, cognitive stress [27] and emotional stress [28]. In this work, we explored the feasibility of implementing a VR-based experimental setup to (1) elicit multifaceted stress response and (2) measure the resulting behavioral and physiological response. Using our validated VR platform *bWell* [29], we aimed to leverage specific exercises among its battery for eliciting different stressor responses, specifically for physical, cognitive, and emotional stress. Particular emphasis was given on designing VR stress exercises to systematically test cardiac functioning within specific contexts of self-regulation (executive functioning and emotional regulation) based on neurocardiac models [30,31]. In addition, by integrating VR with PPG and electromyography (EMG), we captured various physiological measures, including HRV and facial expressions (collected via EMG), along with subjective ratings of stress (namely, the Perceived Stress Scale scores for overall stress and NASA-Task Load Index [NASA-TLX] for specific stress dimensions) and mood.

The overarching goal is that the study design or paradigm tailor to different stress domains and measurable behavioral and physiological outputs. The long-term goal is to perform multivariate and ML-based analysis for predicting the subjective perceived stress of an individual. In addition, it should be adaptable to the specific needs of each individual, allowing a systematic and individualized assessment of stress response.

## Methods

### Development Process

Because the work involved immersive VR in a health context, the “Recommendations for Methodology of Virtual Reality Clinical Trials in Health Care by an International Working Group” were followed during the development process [32]. Following the recommendations, we focused on the first two phases: (1) VR1 phase with an emphasis on content development of different VR stress exercises and (2) VR2 phase with an emphasis on verification trials testing feasibility, acceptability, and tolerability. In addition, the study development process also followed guidelines from the Medical Research Council, namely *“*Guidance on how to develop complex interventions to improve health and healthcare,*”* [33] which recommends combining the opinions or input elicited during content development with existing or available theories (eg, the rationale for intervention, expected changes, and how changes may be achieved). On the basis of the guidelines, our development study included a precursor step to identify the prevailing theories and frameworks in the domain to formulate hypotheses for the study.

**Figure 1:**
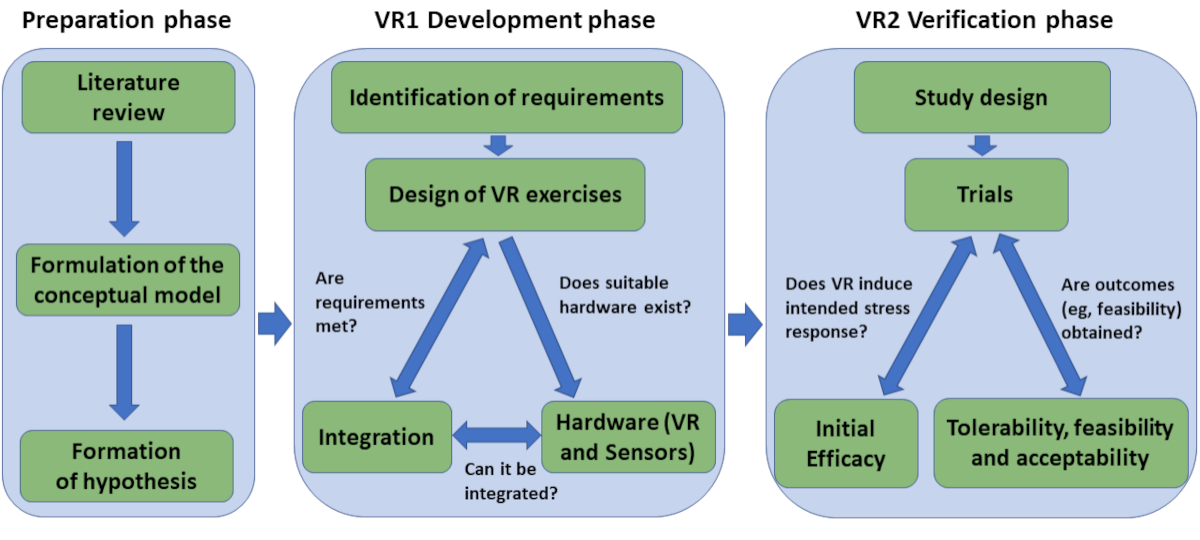
Schema of the development process. The development process comprised three phases: preparation phase, virtual reality (VR)-1 development phase and VR-2 verification phase.

Consequently, our development process comprised three phases: (1) a *preparation phase,* during which an extensive literature review was performed to establish the links between heart, brain, and stress, leading to the formulation of a conceptual model and formation of hypotheses (precursor phase); (2) a *development phase,* which involved iterative design of VR exercises for targeted stressors and hardware integration (VR1 phase); and (3) a *verification phase* (VR2 phase), focusing on implementing a workflow able to test the outcome measures associated with VR2 trials—tolerability, acceptability, and feasibility (Figure 1).

### Preparation Phase

#### Literature Review and Survey

A literature survey was performed to establish links between stress, the heart, the brain, and cognition. Given the involvement of several bodily systems in stress, we observed diverse findings: on the one hand, associations with mental load [34] and emotional stress [35], on the other hand, associations with cognitive performance [36,37]. With the goal of linking these diverse findings, we next focused on publications that have reported integrated models of the heart, the brain, and cognition (especially self-regulation, a cognitive process implicated in stress). The first such model is the *Neurovisceral Integration Model* *(NVIM)* [38,39]. According to the NVIM, the heart and the brain are connected by direct and indirect pathways that influence the various processes (physiological, behavioral, emotional, and cognitive) related to self-regulation and adaptability. More importantly, the NVIM postulates vagal tone as the common index that is linked to physiological, affective, and cognitive regulation. The second model is based on the *Vagal Tank Theory (VTT)* [31]. The VTT suggests that a higher vagal tone is associated with better self-regulation and adaptive functioning.

It is worth noting that the theories of interest focus on vagal tone, which refers to the activity of the vagus nerve. The vagus nerve is the largest autonomic nerve, innervating nearly every organ in the body. More importantly, the vagus nerve regulates metabolic homeostasis by controlling the heart rate [40] as well as the interval between 2 heartbeats (HRV) [41]. While direct measurement of vagal activity (ie, physiological studies of the vagus nerve) has been limited due to difficulties in recording chronic signals in the vagus [42,43], HRV provides an indirect measure of vagal tone (a clinical metric to specify overall levels of vagal activity) because it is largely regulated by cardiac vagal control [44,45]. HRV has been shown to be a sensitive metric for detecting abnormalities in vagal tone, as observed in clinical conditions such as diabetes, heart failure, and hypertension [46-48]. More broadly, HRV, as an indirect measure of vagal tone, has shown to be useful in many aspects of psychophysiology, such as self-regulation mechanisms linked to cognitive, affective, social, and health phenomena [13,30,49]. In summary, HRV is commonly used in research as a practical index for vagal tone, and therefore, we will be using HRV in our study to link vagal tone with stress.

#### Formulation of the Conceptual Model

To formulate our model, we first examined the hypotheses of the NVIM and VTT. Specifically, the NVIM suggests that greater parasympathetic nervous system activity (ie, vagal tone) increases regulatory control in the brain and body [38,39]. This control supports certain biological processes such as digestion, while facilitating brain functioning related to cognitive control and emotional regulation. In contrast, withdrawal of vagal tone supports physical action, such as a fight-or-flight response. The parasympathetic nervous system is thought to monitor and balance feedback from numerous subsystems to determine the optimal level of vagal tone to deploy, given one’s current context, active goal, and unmet needs [50]. HRV reflects vagal tone and, as such, should correlate with the integrity of cognitive and emotional control processes [30]. By contrast, VTT suggests that there are *3* states of vagal control, the “3 Rs”: *resting, reactivity,* and *recovery* [31]. Optimal vagal tone is high at rest, so a healthy system should settle to a high baseline level of parasympathetic activity. In response to mental demands, vagal control systems should react by maintaining a high vagal tone to promote executive control. In response to more active demands, these systems should react by withdrawing vagal tone to promote physical performance. In practice, vagal tone often drops below resting levels even during cognitive tasks, possibly due to the strain on and gradual exhaustion of vagal control systems. Once demands are met, vagal control should transition into a recovery state, where vagal tone returns to resting levels.

The NASA-TLX provides a formal framework for categorizing different aspects of workload and task demands [51]. Therefore, we identified this index as a useful tool for understanding the demands that vagal control systems must continuously monitor and react to, as per the *VTT* and the *NVIM*. Specifically, the NASA-TLX defines 3 types of task demands—*mental*, *physical*, and *temporal*—and 3 additional factors—*performance*, *effort*, and *frustration*—that contribute to perceived workload. On the basis of the *NVIM* model of vagal control, *mental*, *physical*, and *temporal* task demands should be associated with different optimal levels of vagal tone and, therefore, different states of reactivity. *Mental* demands are best solved by promoting executive control through high levels of parasympathetic nervous system activity; in contrast, active *physical* and *temporal* demands are best addressed by a withdrawal of vagal tone.

#### Formation of Hypothesis

On the basis of previous publications, we formed specific hypotheses for self-regulation for each level of cardiac vagal control (Table 1). At *resting (baseline)*, we hypothesized that the higher the vagal tone, the better the self-regulation [49,52-54]. For *reactivity*, different hypotheses were formed for emotional, physical, and cognitive demands. It may be noted that for consistency with terminology in neuroscience, we have used cognitive demand as representing mental demand and emotional demand as representing frustration in the NASA-TLX. For emotional demand, (1) a complete vagal withdrawal would elicit a fight-or-flight response [52,55], (2) a small increase in vagal tone would indicate successful emotion regulation [53,54], and (3) a small decrease would indicate that the task did not elicit a sufficiently large stress response [49,56]. For cognitive demand, (1) we hypothesized an initial increase in vagal tone under low cognitive load (self-regulatory effort) and a decrease in vagal tone under high cognitive load [31,57], and (2) in the context of vagal withdrawal (ie, high cognitive load), we hypothesized that a small decrease in vagal tone would predict better performance [31,58]. For physical demand, a small vagal withdrawal would indicate quick adaptation to meet the demands of the situation [59,60]. Finally, for *recovery*, we hypothesized that the faster the vagal tone returns to the baseline level, the better the self-regulation [61].

**Table 1:** Description of the hypotheses corresponding to our conceptual model based on the Neurovisceral Integration Model (NVIM) and Vagal Tank Theory (VTT). A distinct hypothesis was made for each phase—resting (baseline), reactivity, and recovery.

Cardiac vagal control level		Hypothesis of self-regulation, as indicated by cardiac vagal control
1. Resting (baseline)		A higher vagal tone indicates better self-regulation
**2. Reactivity**		
	2a. Emotional demand	Complete vagal withdrawal indicates that a fight-or-flight response was elicited A small increase indicates successful emotional regulation A small decrease indicates that the task did not elicit a sufficiently large stress response
	2b. Cognitive demand	An initial increase in vagal tone under low cognitive load (self-regulatory effort) and a decrease under high cognitive load A smaller decrease (under high cognitive load) is associated with better performance
	2c. Physical demand	Small vagal withdrawal indicates quick adaptation to meet demands of the situation
	2d. Physical and cognitive demand	Combination of hypotheses 2c (vagal withdrawal) and 2b (initial increase under low cognitive load with a more pronounced effect)
3. Recovery (from demand)		The faster the vagal tone returns to the baseline level, the better the coping mechanisms

### VR1: Development Phase

#### Identification of Requirements

The fundamental objective of this work is to harness the affordances of immersive VR to provide new opportunities for practicing stress management skills, particularly the synthetic recreation of scenes that are otherwise too difficult or resource intensive to replicate in real life. The latter is of particular interest in the application toward training stress resilience, a complex and dynamic process. Combined with physiological monitoring, which can help to capture additional information on the user state, the use of VR-based technologies can enable research in support of better understanding the impacts of well-being and individual characteristics, such as mental and psychological resilience.

To study and validate the different parts of our conceptual model, we identified four main requirements:

1. *Requirement 1:* recreation of each level of cardiac vagal control (3 “Rs”: resting, reactivity, and recovery)
2. *Requirement 2:* ability to elicit different types of mental demands
3. *Requirement 3:* ability to elicit physical demand
4. *Requirement 4:* integration of physiological sensing with immersive VR, essential for the integration of HRV measures as an index of vagal tone

#### Design of VR Exercises

We have previously developed *bWell*, an interactive and immersive VR platform designed as a broadly applicable toolkit delivering multisensory tasks, targeting general aspects of cognition and everyday functioning [29,62-64]. For this study, we made use of *3* bWell exercises, namely *Tent*, *Mole,* and *Stroll,* which were adapted to systematically test adaptive aspects of HRV within specific contexts of self-regulation (executive functioning, emotional regulation, and physical demand) and for different levels of cardiac vagal control, as identified in our conceptual model and requirements 1 and 2. The bWell platform and exercise battery are described in detail in our previous publication [29]. The following is a brief summary of the three exercises:

1. *Tent*—an exercise designed for self-guided stress management in which the user is immersed in scenic views and asked to look around while focusing on their breathing
2. *Mole*—an exercise targeting response inhibition and cognitive control in which the user has a hammer in each hand and has to hit cylinders that pop up in front of them (only when the cylinder and hammer are of the same color)
3. *Stroll*—an exercise targeting sustained attention with a continuous performance test (CPT) in which the user has to press a button for each new shape that appears, except when it is a green diamond. The user is represented by a self-avatar and is passively displaced for a stroll in a natural scene.

To test our specific hypotheses for different stress demands or stressors (Table 1), we adapted the aforementioned bWell exercises (except for the *Mole* exercise for cognitive demand) as follows:

1. *Tent*
  - Nature scene to collect data on user
    *Resting:* As per recommended guidelines for experimental settings, baseline HRV should be recorded in a “nontask situation that best controls for the presence of task comparison” [65]. Consequently, the administration of the exercise was modified such that the user was asked to simply immerse themselves in the scene, limit physical movement, and breathe spontaneously for 5 minutes.
    *Recovery:* this scene was also selected to be administered after a given task to allow a period of recovery after stress demand. As a measure of recovery, a Likert scale implemented in VR was added to collect a report on mood right after the exposure to a stressor and after the recovery period in a relaxing nature environment.
  - *City*—in addition to the nature scenes, to elicit emotional demand, we adapted the *Tent* exercise to include an environment such that the user is immersed in a busy city with noise pollution—roads with ongoing traffic, including audio honking cars and sirens.
2. *Stroll*—to exert physical demand, we adapted the Stroll exercise such that the user must physically displace themselves to advance in the scene. The user was asked to jog, keeping their heart rate at a moderate level of exertion (the predefined threshold was displayed as a gauge based on individual heart rate). The exercise could include a CPT, as described earlier, to exert both physical and cognitive demand (*Stroll+CPT*) or could be administered without the test as a purely physical task (*Stroll*).

The formulated hypotheses shown in Table 1 can now be systematically matched with our designed VR exercises (Table 2).

**Table 2:** Description of the hypotheses corresponding to our conceptual model with the designed virtual reality (VR) exercises. A distinct hypothesis was made for each phase—resting (baseline), reactivity, and recovery—and these hypotheses were tested with different VR exercises.

Cardiac vagal control level		Requirement	bWell exercise
1. Resting (baseline): Tent—nature scene, no specific task given		User to immerse themselves, limit physical movement, and breathe spontaneously as a baseline state	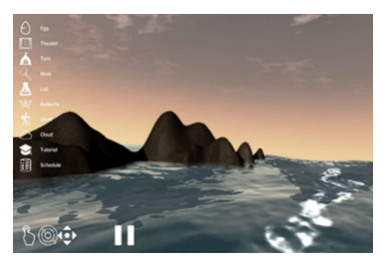
**2. Reactivity**			
	2a. Emotional demand: city—(passive) city scene	User to immerse in a busy city with excessive noise pollution to elicit emotional demand	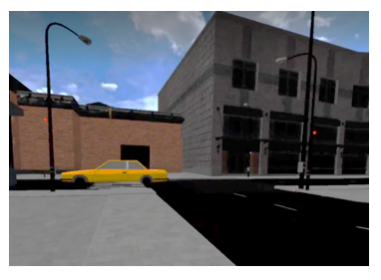
	2b. Cognitive demand: mole—(active) targeting response inhibition	User to engage in a dynamic and bimanual go or no-go task to elicit cognitive demand	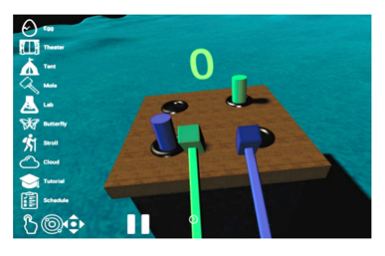
	2c. Physical demand: stroll—(active) physical running task	User to jog to create a physical demand, with heart rate biofeedback guiding them to maintain a consistent moderate level of activity	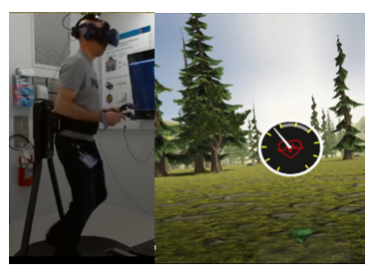
	2d. Physical and cognitive demand: stroll+CPT^a^−dual task with running task as 2c along with a cognitive task (response inhibition)	User to jog and engage in the task to elicit both physical and cognitive demands	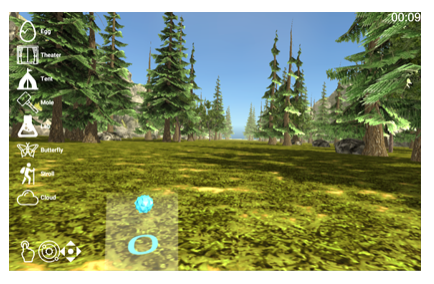
3. Recovery from demand: Tent—nature scene		User to immerse themselves and limit physical movement to recover	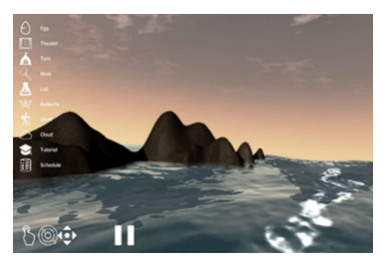

^a^CPT: continuous performance test.

#### Hardware Identification (VR+Sensors)

Based on the identified requirements, we aimed to identify and integrate hardware for use with the bWell exercises. We focused on evaluating the suitability of the use of immersive VR technologies. The choice of hardware considered specialized equipment aiming to maximize the feeling of “*presence*” in virtual environments as well as the potential to democratize the use of VR for training more broadly. Consequently, we evaluated both a high-end system—*HTC Vive Pro Eye*—or use within dedicated facilities as well as a more accessible option—*Pico Neo 3 Eye*. The latter targets the opportunity for individuals to practice at home or in settings outside the laboratory (eg, on the field, classroom, and clinical).

We aimed to reproduce the required factors in the simulated environment with the integration of specialized hardware. A VR treadmill (KatVR) was identified to recreate the physical demands. We also aimed to capture stress via the inclusion of physiological sensing, with a focus on integrating heart-based measures. The *EmteqPRO* facemask was identified for use in this project because it is a commercially available “plug-and-play” system for use with immersive VR, provides researchers with access to raw data, and is equipped with automatic classification algorithms for processing raw heart rate data into HRV as well as classification of emotion [66]. The system can accommodate multimodal measures of EMG and HRV via the use of a facemask that can be directly integrated onto the VR headset. The Emteq device does not add any additional steps to the workflow for carrying out trials with immersive VR; instead, the user simply must put on the headset as they normally would.

#### Hardware Integration

A first evaluation was conducted to determine the suitability of the identified hardware.

#### Immersive VR Headset

The *HTC Vive Pro Eye* and the *Pico Neo 3 Eye* headsets were identified because they both offer eye tracking. In addition, the Emteq solution is available for both. The HTC Vive Pro Eye is a tethered device, meaning it requires a high-performance, VR-ready PC to operate. A tethered VR headset operates only when connected to a PC through a cable connection and offers superior graphics and fidelity in comparison to stand-alone headsets. The *Pico Neo 3 Eye* is a stand-alone headset, which is wireless, and the VR is run entirely within the headset. The benefits of a stand-alone headset include increased comfort and mobility, a simple setup, and a lower cost compared to tethered devices. However additional efforts to optimize the VR experience in terms of graphical rendering and design are required to minimize lags related to hardware performance.

It was determined that both headsets are suitable for use in the proposed study with certain adaptations. A wireless adapter was integrated into the HTC Vive Pro Eye, enabling untethered use with the treadmill. For the Pico Neo 3 Eye, an external dongle was required to enable screen casting to an external monitor, allowing the test administrator to view the participant's perspective within the VR during trials.

#### Emteq Integration With bWell in Unity

**Figure 2:**
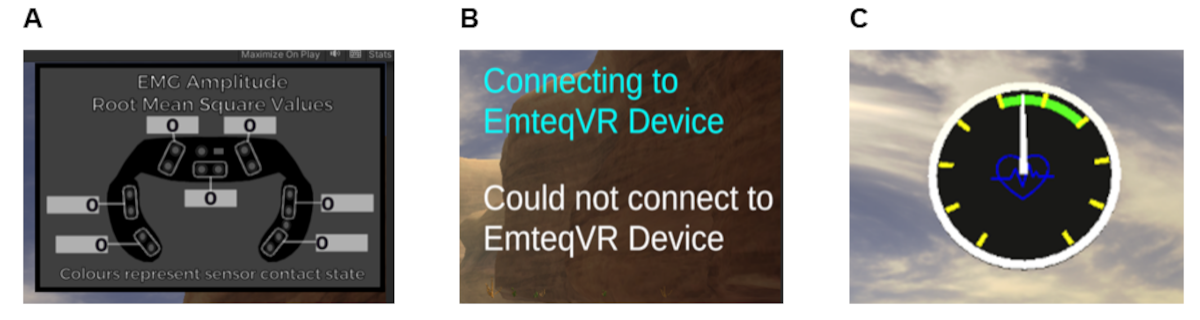
Integration of physiological sensor with virtual reality (VR). Implementation of widgets in the user interface for integration of EmteqPRO (physiological sensor for heart rate variability) into VR platform (bWell): (A) widget for displaying electromyography (EMG) amplitude in VR, (B) widget for connection status of EmteqPRO, and (C) widget for displaying heart rate.

The Emteq face mask was integrated within *bWell* through the use of the “EmteqLabs Unity” plugin. Using the plugin, the EMG amplitude as well as the heart rate (beats per minute) of each participant was extracted. Using these extracted values, several widgets (Figure 2) were implemented in the 2 existing layers of the *bWell* user interface (UI): test administrator UI and participant UI. The first widget was part of the EmteqLabs plugin package and was used to display EMG amplitude (Figure 2A). The same widget also provided the administrator with an indication of sensor contact. Good contacts were represented by green dots on the widget, while bad contacts were represented by gray dots. The second widget was composed of 2 simple text boxes that were used to inform the test facilitator of the current connection status of the Emteq (Figure 2B). The third widget was a speedometer that was used to inform the participant whether they were in a heart rate zone of moderate effort (Figure 2C). The widget was implemented as a gauge to prompt participants to raise or lower their effort level, with the zone in the widget turning green when participants are at the correct range. Specifically, (zone turns green at correct range). A heart icon that grew and shrank based on a speed that matched the current beats per minute was also included in the speedometer.

#### Treadmill Integration

The usability of donning a wireless immersive VR headset while running on the treadmill was tested. Running speed (vigorous vs moderate) was evaluated, and it was found that both vigorous and moderate running was acceptable in terms of usability for both headsets. However, it was seen that running on a treadmill was not intuitive and that a warm-up time was required. A test run was added to the protocol, such that the user could have some time to familiarize themselves with running on the treadmill before putting on the VR headset.

It was also seen that vigorous running may prove to be difficult for participants running on the treadmill for the first time. In addition, running on the treadmill while performing a cognitive exercise (*Mole* exercise) occasionally resulted in inadvertent errors from the user. As a result, the protocol was adjusted to have users run at a moderate pace (light jog) during the study. For the dual task (Stroll+CPT), a cognitive exercise with simpler user interaction was use. The exercise was changed from the initial choice to use *Mole* (requiring users to swing with their arms) to the *Stroll* (requiring users to pull a trigger on the controller).

Finally, the physical demand exercise (*Stroll*) was originally designed for passive displacement. In this task, it is the self-avatar taking a nature stroll while the actual user is not moving. To promote a higher sense of *presence* for the user, we added advancement in the VR scene based on the user’s own steps while advancing on the treadmill. The running pace was added as an output measure in the VR logs generated automatically after a session with bWell.

The conclusion from the hardware integration tests was that both the *HTC Vive Pro Eye* and *Pico Neo 3 Eye* were suitable. However, because the primary use at this stage was for study purposes, it was decided that it would be better to use the hardware that is designed for external administration of VR versus self-administration with a headset. In the workflow for administration, the bWell platform provides a UI that allows the test facilitator to dynamically change the exercise settings while a user is immersed in VR [29]. Consequently, it would be better suited for the iterative approach in finding the optimal exercise settings for the study. Thus, the decision was to use the *HTC Vive Pro Eye* for the study and the ensuing verification trials.

### VR2: Verification Phase

In this phase, we focused on conducting early trials for evaluation and verification of feasibility, acceptability, tolerability, and initial efficacy (VR2 phase).

#### Study Design

**Figure 3:**
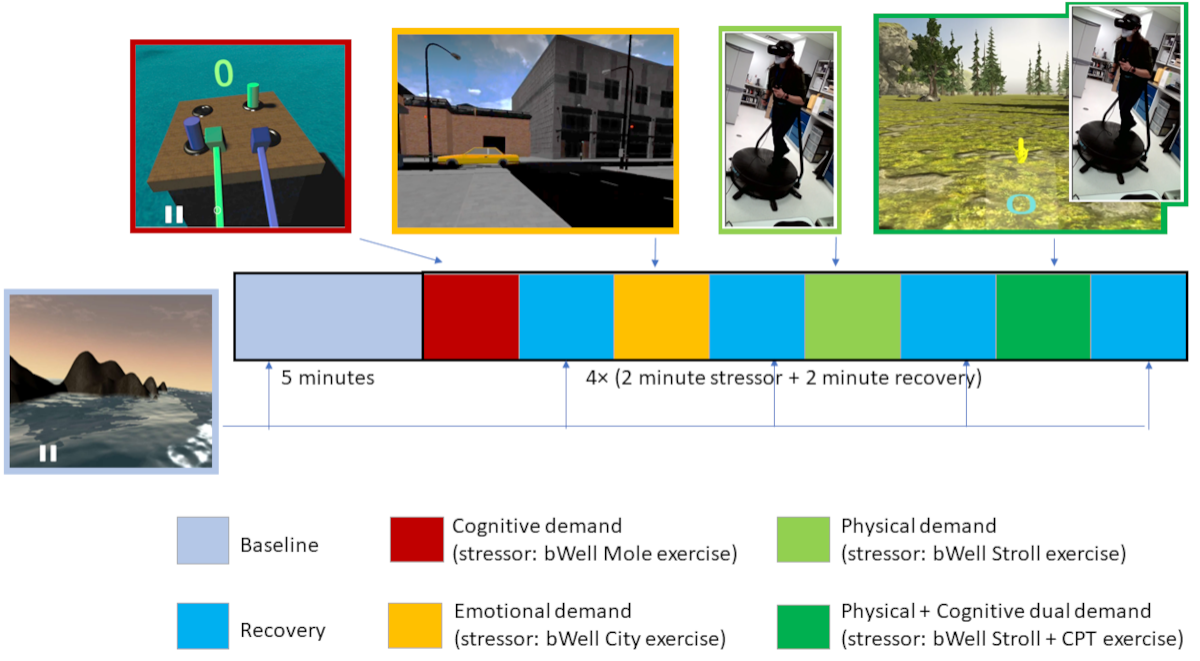
Description of the study design. A within-participants repeated measures study design was implemented. It comprised an initial baseline (5 minutes), followed by 4 blocks of stressor (2 minutes each) and recovery (2 minutes each) periods. The stressors were 4 virtual reality (VR) exercises targeting emotional demand, cognitive demand, physical demand, and cognitive+physical demand. The sequence of stressors was pseudorandomized. CPT: continuous performance test.

We designed a study to systematically test our hypotheses (Figure 3). The study protocol consisted of a 5-minute initial baseline consistent with what is used in the HRV literature (Table S1 in Multimedia Appendix 1). During baseline, participants were in a standing position with their eyes open to optimally match the body position used during task conditions. Participants experienced a neutral nature scene. The baseline HRV was collected to establish a reference point. Participants then experienced alternating blocks of stressor tasks interspersed with recovery (Tent nature scenes). Stressor task presentation order was pseudorandomized across participants. Each participant performed all 4 exercises (stress conditions). Each stressor condition was followed by a 2-minute recovery period in the *Tent* (Table S1 in Multimedia Appendix 1).

The measures of VR2 trials were captured as follows:

- *Acceptability:* user experience, as assessed by the game user experience satisfaction scale (GUESS)-18 gamer user experience satisfaction scale [67] and the System Usability Scale [68]
- *Tolerability:* user comfort and any side effects from the use of technology, as assessed by the Simulator Sickness Questionnaire (SSQ; captured both before and after VR exposure)
- *Feasibility:* ability to elicit the intended stress response, as measured via physiological response as well as self-report

For the self-report measure, the NASA-TLX [51] and single-item rating for stress questionnaires were used.

Mood Likert scales were also automatically administered in immersive VR before and after the recovery exercise. Participant and VR views were also recorded. After completing the bWell session, participants reviewed a screen recording of the completed virtual task, provided qualitative feedback, and completed the set of questionnaires.

It may be noted that while the NVIM and VTT predict the dependence of vagal tone on task conditions, the NASA-TLX measures 6 aspects of workload. The study design involved 5 conditions, 1 without a stressor and 4 others in which each condition features a different exercise representing specific stressors based on the NASA-TLX (summarized in Table 3). To effectively assess the effects of each condition on stress responses, we proposed a within-*participants* design, where each participant completed all conditions of the experiment. The order of the stressor conditions was pseudorandomized across participants. The approach taken to experimentally isolate the contributions of each type of stress required the removal of performance criteria (eg, score) from the tasks, as they may create confounds in the stressor type not related to the experimental manipulation of the condition. However, the cumulative effects of stressors were also investigated using a physical+cognitive stressor dual task, as the inclusion of multiple simultaneous stressors is more representative of everyday stressful situations.

**Table 3:** Description of the different types of stressor demands for different virtual reality (VR) exercises. bWell VR exercises elicited different types of demands, as defined by the NASA-Task Load Index workloads. The main stress or demand captured for each VR exercise is shown in italics.

	Mental demand	Physical demand	Temporal demand	Performance	Effort	Frustration
Relaxing nature environment (*tent*: nature scene)	No	No	No	No	No	No
Emotional stressor (*city*: busy city scene)	No	No	No	No	No	*Yes* ^a^
Cognitive stressor (*mole*: dynamic bimanual go or no-go task)	*Yes* ^a^	No	Yes^a^	Yes^a^	Yes^a^	No
Physical stressor (*stroll*: running task)	No	*Yes* ^a^	No	Yes^a^	Yes^a^	No
Physical+cognitive stressor (*stroll+CPT:* running with continuous performance test)	*Yes* ^a^	*Yes* ^a^	No	Yes^a^	Yes^a^	No

^a^Demands elicited by the bWell virtual reality exercises.

We set thresholds to the NASA-TLX as a self-reported measure to ensure that the designed VR exercises targeted the intended type of demand, that is, *cognitive, emotional, physical,* and *dual (ie, physical+cognitive)*. We defined the following threshold in terms of NASA-TLX score (score range=0-10): a score>5 indicates that the demand is being elicited (eg, *City* for emotional demand, *Mole* for cognitive demand, *Stroll* for physical demand, and *Stroll+CPT* for physical and cognitive demand; Table 4). An additional aim was to not elicit demand related to poor performance because this may lead to a potential confound. In the NASA-TLX, performance is reverse coded (ie, 0=perfect performance). As such the threshold was set to<4, so that that participant should feel as if they performed well in all exercises. Finally, since all exercises invoked a type of demand except the one involving emotional demand, they were expected to involve an element of effort.

**Table 4:** Description of the thresholds for NASA-Task Load Index (NASA-TLX) scores for eliciting different stress demands. We set the NASA-TLX subscale scores to above the threshold, for the different types of stress demands that were elicited by each virtual reality exercise in Table 3.

	Frustration	Physical demand	Mental demand	Performance
City	>5^a^	—^b^	—	<4
Mole	<4	<4	>5^a^	<4
Stroll	—	>5^a^	—	<4
Stroll+CPT^c^	—	>5^a^	>5^a^	—

^a^NASA-TLX subscale scores that were above the threshold.

^b^Not available.

^c^CPT: continuous performance test.

More specifically, we targeted the following scores:

- *City*—a NASA-TLX score of >5 for *frustration,* which would indicate an emotional demand. We also intended for all other NASA-TLX scores to remain <4 to solely evoke an emotional response. Given the passive nature of this exercise, other demands should not be induced.
- *Mole*—a NASA-TLX score of >5 for *mental,* which would indicate a cognitive demand. A score of >5 would indicate that these demands were elicited. We also targeted *frustration* and *physical* NASA-TLX scores of <4 to solely target demands related to cognitive performance.
- *Stroll*—a NASA-TLX score of >5 for *physical,* which would indicate a physical demand. A demand of >5 would indicate that this was the case. We also targeted *mental* and *frustration* NASA-TLX scores of <4 to solely target demands related to physical performance.
- *Stroll+CPT*−a NASA-TLX score of >5 for *mental* and *physical,* which would indicate a dual cognitive and physical demand. We also intended to maintain a *frustration* score of <4 because we wanted to target solely cognitive and physical demands.

In addition, we sought to verify that *mental* demands are comparable between the *Mole* and *Stroll+CPT* tasks.

### Verification Trials

Verification trials were conducted using HTC Vive Pro Eye on 3 healthy participants according to the protocol defined during the study design.

### Ethical Considerations

Ethics approval for the study was obtained from the Research Ethics Board (REB) of the National Research Council (NRC 2020-2023 and NRC 2023-46) and from Defence Research and Development Canada Human Research Ethics Committee (HREC 2022-032). Informed consent was obtained before the start of the study. Data were deidentified before any analysis. There was no compensation paid to the participants.

## Results

The results of the verification trials are reported below in terms of tolerability, acceptability, and feasibility.

### Tolerability: Cybersickness During the VR Sessions

We first checked whether the VR exercises designed in our study induced cybersickness using SSQ [69]. Table S2 in Multimedia Appendix 1 shows the scores for SSQ for pre- and post-VR sessions for 3 participants. Total SSQ scores were obtained from the scores of the 16 items of the questionnaire [70]. Results showed negligible score (total SSQ score: 0-5), which are within the acceptable range in the literature [71]. Thus, our results show that the participants did not experience adverse effects, especially cybersickness during the VR exercises.

### Acceptability: User Experience

**Figure 4:**
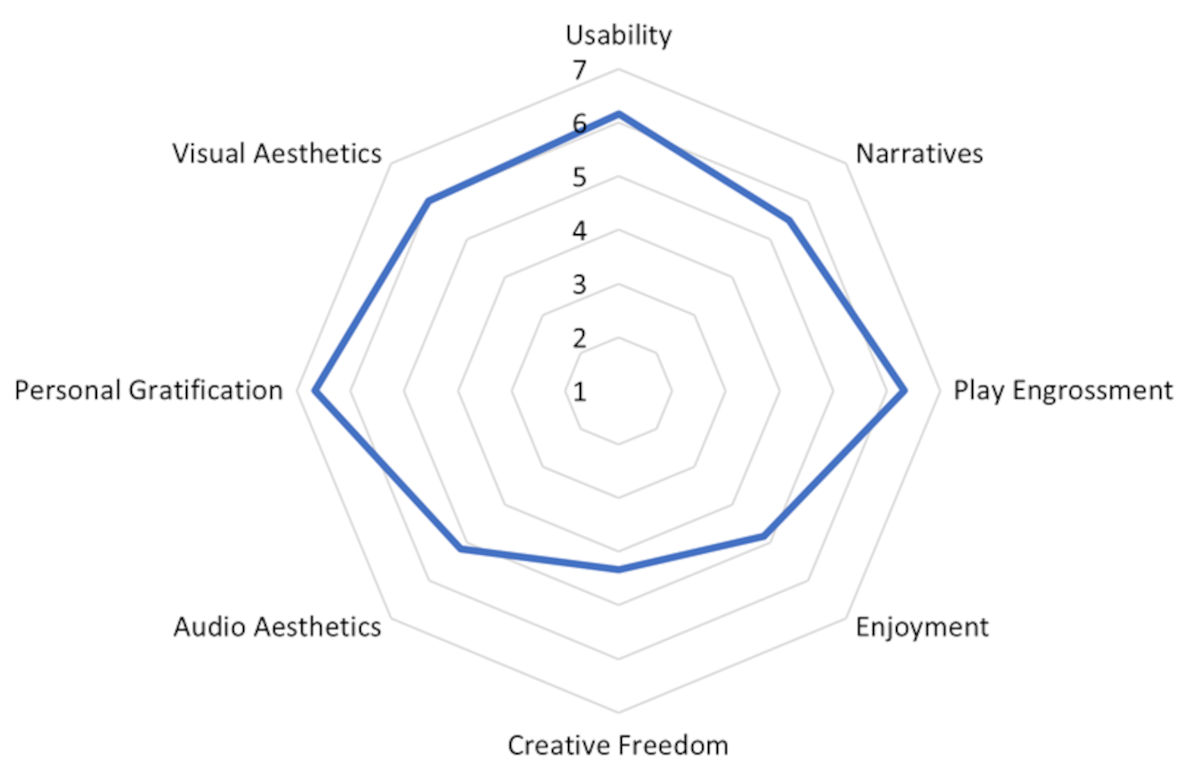
Findings of user acceptability. User ratings on the game user experience satisfaction scale (GUESS)-18 questionnaires showing greater average scores for the subscales usability, play engrossment, and personal gratification and lower average scores for creative freedom and enjoyment. An overall GUESS score of 43.17 (Table S3 was observed, which aligns well with previous findings, indicating the usability and immersiveness of the virtual reality exercises designed in our study.

Using the GUESS-18 scale [67], we checked whether the VR exercises designed in our study were immersive. GUESS-18 has 9 subscales with 2 statements associated with each subscale. Of the 9 subscales, we did not include “social connectivity,” as there was no interaction between participants for the VR exercises. Table S3 in Multimedia Appendix 1 shows the scores of the 8 subscales of GUESS-18 for the 3 participants. The subscale scores of the GUESS were computed by averaging the scores within each subscale. Then, the overall GUESS score was computed by summing the subscale scores [67]. We observed an overall GUESS score of 43.17 (Table S3 in Multimedia Appendix 1), which aligns well with previous findings [72] and indicates the usability and immersiveness of the VR exercises designed in our study. Regarding the individual subscales, we observed greater average scores for *usability*, *play engrossment,* and *personal gratification* and lower average scores for *creative freedom* and *enjoyment* (Figure 4).

### Feasibility: Do the VR Exercises Induce Genuine Stress Responses (Perceived)?

**Figure 5:**
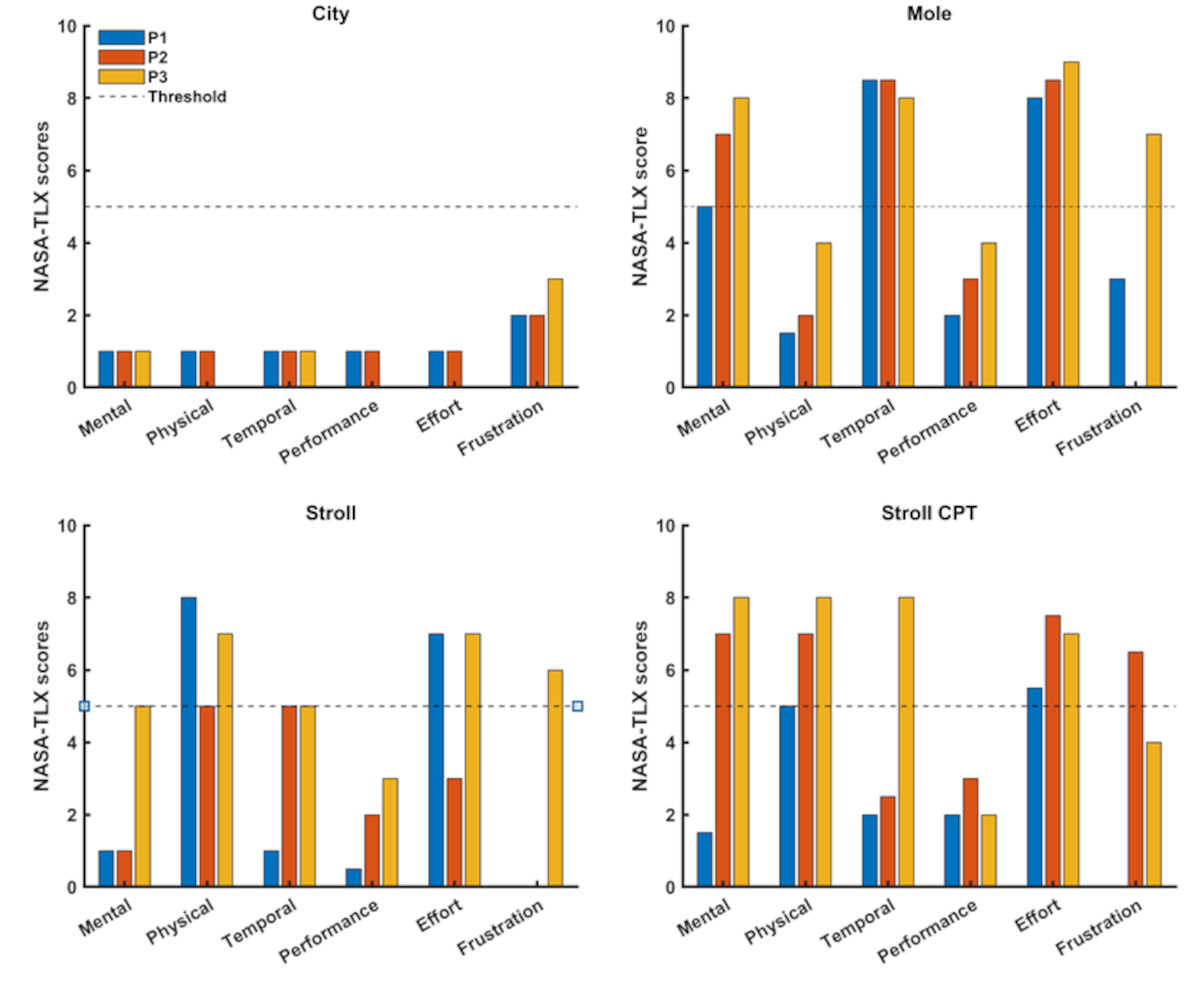
Comparative analysis of different types of demands for different stressors. User ratings on the NASA-Task Load Index (NASA-TLX) revealed that participants perceived different types of demands (eg, mental, physical, and temporal) for the different VR exercises. Here, P1, P2, and P3 denote participant 1, participant 2, and participant 3, respectively, and the dashed line represents the threshold set for different demands associated with the different stressors (exercises). CPT: continuous performance test.

We evaluated the NASA-TLX scores to investigate the types of stressors and their associated demands. Figure 5 summarizes the data for NASA-TLX from verification trials of 3 participants.

On the basis of the designed thresholds for the different stressors and their associated demands (refer to the Methods section), we observed the following for each of the bWell exercises (Figure 5), which led to further iterative development:

- *City*: a NASA-TLX rating of >5 for *frustration* was not achieved, indicating a need for adapting the exercise settings and creating new functionalities. All other demands were also not elicited (<4). In particular, users felt that they performed well (<4), and as such, it was verified that there would not be any confounds in *frustration* being elicited due to emotional response related to feelings of poor performance rather than VR stimuli.
- *Mole*: a cognitive demand was elicited with a *mental* demand of >5. We also observed a high level of *temporal* demand and associated *effort*.
- *Stroll*: a *physical* demand and associated *effort* was elicited (>5).
- *Stroll+CPT*: a dual cognitive (*mental*) and *physical* demand was elicited (>5), along with associated *effort*.

Because cognitive demand was elicited in both tasks, similar to the Mole task, the 2 can be compared in the analysis of cognitive demand, even though they involve different cognitive tasks. It appears that *temporal* demand may also be elicited but was not conclusive at this stage because of high variability in the ratings. This may be clarified with results from additional participant trials from the full study. From Table 4, we observed that this demand was not consistently present. This may be attributed to the fact that this was a dual task, where the *temporal* demand may be felt for one aspect (eg, running) but not for the other (eg, cognitive task), and when prompted by the NASA-TLX, the participant may respond with only 1 task in mind.

Overall, the results showed that we achieved eliciting different stress demands as intended in our study design: for *Stroll* to exert a *physical* demand, *Mole* to exert a *mental* demand, and a combination of exercises (*Stroll+CPT*) to exert both *mental* and *physical* demands. At the same time, we observed that participants felt they performed well in all exercises, minimizing confounds due to feelings of poor performance. These verification trials allowed us to finalize the exercise configurations to be used for the full study trials.

### Feasibility: Do the VR Exercises Induce Genuine Stress Responses (Physiological)?

To evaluate the physiological stress responses, we examined the HRV measures obtained, specifically the root mean square of successive differences (RMSSD) between normal heartbeats. We adopted RMSSD as the HRV measure because previous studies have shown that RMSSD reflects vagal tone [38,73] and is relatively free of respiratory influences. It was seen that the data were quite noisy, artifacts were present, and the RMSSD values from the raw PPG data were too high. For example, values in the range of 150 to 300 were obtained, which is much higher than normal. Discussion with the Emteq team revealed that this was likely because the measures were sampled in a dynamic VR environment, particularly where in some instances, users were physically running during PPG data acquisition. Emteq advised using their signal quality metric and using only the data where good quality was indicated.

To have better control of the data processing, the team decided instead to implement an in-house data processing pipeline. For this, the raw PPG signals were processed using the Python library *HeartPy* [74]. On the basis of the *HeartPy* documentation, which states that RMSSD is extremely sensitive to incorrectly detected heartbeat locations, any RMSSD value artifacts (RMSSD does not typically exceed 130) were removed and peak interpolation was used to minimize the number of incorrectly placed peaks due to low sampling rate [74]. After these steps, an improved signal-to-noise ratio was observed and the resultant RMSSD values fell well within the normal range expected (between 30 and 70).

**Figure 6:**
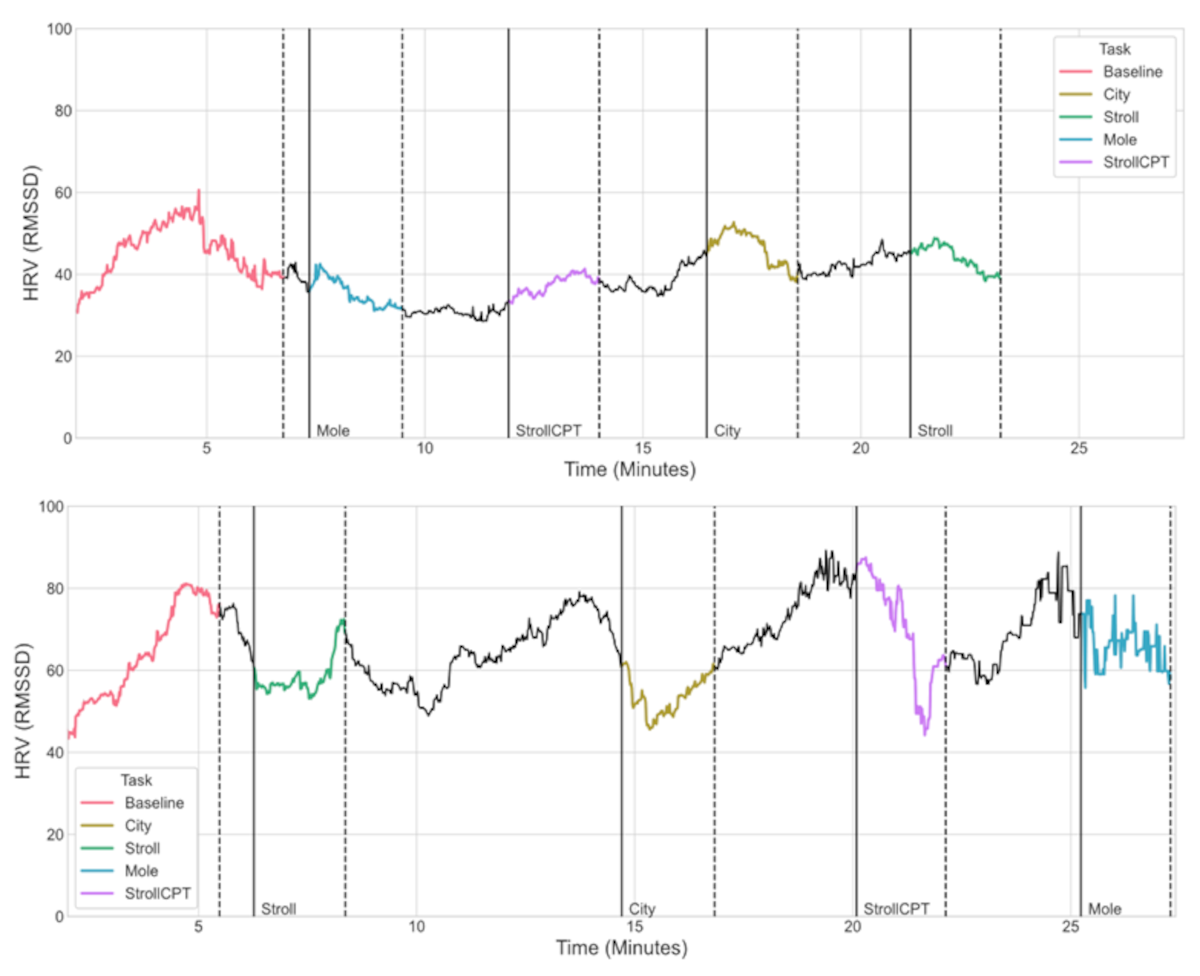
Characterization of distinct patterns of heart rate variability (HRV) for different virtual reality (VR) exercises. HRV data (root mean square of successive differences [RMSSD]) for the different VR (bWell) exercises are shown for 2 participants (row 1 for participant 1 and row 2 for participant 2) along y-axis and time along x-axis. The plots illustrate distinct HRV responses for the different exercises as well as individual variability in the HRV responses. CPT: continuous performance test.

We then compared HRV across each of the different conditions for 2 individuals. In the example shown in Figure 6, it can be seen that a given task appears to elicit a change in response. It is also evident that baseline, reactivity, and recovery HRV patterns were distinct for the 2 sample individuals. As can be seen in the lower panel of Figure 6, some instances of residual noise remain in the Mole exercise after processing. Given that the data are being collected during interactive tasks with a good degree of movement, a high noise-to-signal ratio was expected. The next steps will involve exploring other techniques, particularly for motion artifact removal, with the goal of further reducing noise in the signal.

## Discussion

### Principal Findings

In this paper, we described the development process of a study design for testing the ability of immersive VR exercises to elicit genuine stress responses. The study development followed recommendations for use of VR in clinical trials [32] as well as guidance in developing complex interventions for improving health care [33]. On the basis of these guidelines, our study comprised a combination of *prevailing theories and frameworks* (eg, NVIM and the VTT), *experiences of users,* and *iterative actions during development,* which resulted in concrete outcomes [33]. More specifically, we built on our validated VR platform *bWell* [29] to integrate physiological sensors (PPG) and assessed how different types of VR exercises target different types of stressors (characterized by the NASA-TLX). The results of verification trials showed that the designed VR exercises induced negligible cybersickness, were well tolerated, and demonstrated high user engagement. In addition, in alignment with our hypotheses, the different VR exercises successfully elicited the intended stress demands (as measured with NASA-TLX subscales, eg, mental, physical, and temporal demands), along with distinct physiological responses.

In line with the recommended guidelines for VR trial design in the health domain [44], our study design considered both tolerability and acceptability. Keeping in mind user comfort during VR, the exercises were designed in such a way that the duration for the entire session lasted only 25 minutes. In addition, during the VR session, every exercise was followed by a 2-minute recovery state. By allowing the participants to experience alternating blocks of stressor tasks interspersed with recovery, this study design was used as a guide to evaluate the technical feasibility and scope for the phase of verification trials. Indeed, the results of the verification trials showed user comfort and tolerability, especially VR as an acceptable framework with *negligible* experience of cybersickness (assessed with SSQ scores; Table S2 in Multimedia Appendix 1). As a measure of acceptability, results of verification trials using the GUESS-18 scores showed a high degree of “*presence*” during our VR sessions (Table S3 in Multimedia Appendix 1 and Figure 4), consistent with earlier findings [72]. Because the sense of “presence” (or the subjective feeling of “being there”) characterizes the experience of users, results showing a high degree of presence indicate that the VR exercises designed for the study created the sensation of physical presence and resulted in users behaving and reacting as in the real world, highlighting the importance of a good user experience and engagement. Best practices for HRV data collection were also considered for the study design. In experimental settings, it is recommended that the baseline HRV should be recorded in a “nontask situation that best controls for the presence of task comparisons” [65]. Consequently, in this study, baseline was recorded in VR and in a similar position to how the participants would complete the task. Specifically, baseline was recorded with the user on the treadmill and passively experiencing the VR environments. The session to collect baseline HRV was designed as follows: eyes open, standing, spontaneous breathing, and free to look around and immerse themselves in the VR environment. In addition and according to guidance on HRV reporting, RMSSD was selected as the HRV measure because it is appropriate for short sampling times (ie, <5 minutes) [41].

While the results of verification trials determined the feasibility and validity of our study protocol, some results indicated that modifications were also required. For example, the NASA-TLX scores showed high levels of *Frustration,* which captured participants’ physical demands or frustration related to performance and not necessarily due to an emotional stressor. Because our original goal was to investigate the effects of emotion, we made certain modifications in our bWell exercises to elicit emotional responses in the participants, such as amplifying the noise pollution in the virtual environment. The results of the verification trials also indicated that we need to modify the administration of the NASA-TLX, which currently probes *Frustration* related to both performance and emotion. Instead of asking *“*How insecure, discouraged, irritated, stressed and annoyed were you,*”* the question will be modified to include only the elements related to emotion “How irritated and/or annoyed were you?”

### Implications

It may be mentioned that the overarching aim for the integration of behavioral and physiological outputs was to eventually enable systematic and individualized assessment of stress response. Responses to stress are often multifaceted with a profile specific to the individual. A given stressor may cause a response in one person but not necessarily in another, which may be attributed to the influence of personal experiences. The result is that everyone does not experience stress in the same way. In an effort to standardize the demands placed on individuals, we relied on several mechanisms to adapt the VR exercises in real time and customized them to the participant. For cognitive demand, we aimed to elicit a uniform cognitive demand among varied individuals. To do so, we leveraged the adaptive algorithms in *bWell* to control the difficulty level based on performance (for *Mole* exercise). The adaptive mechanism is designed to maintain an 80% success rate, ensuring the exercise is neither too easy (boring) nor too difficult (demotivating). For physical demand, we made use of the heart rate biofeedback to ensure consistent physical demand because running at a specified speed may be more or less exerting for one individual versus another. To achieve this, a heart rate gauge was displayed to the participant to encourage them to maintain a level of physical activity that corresponds to moderate intensity. This measure is additionally tailored to the individual because it considers the age of the participant, where moderate physical activity is calculated at 50% to 70% of maximum heart rate (maximum heart rate=220 – age).

### Study Limitations

There were certain limitations in our study. First, participants reported issues with disengagement and a lack of frustration during the VR exercise (city). The reason for this could be the low resolution of the visual assets in the scene, a concern that will be addressed in future work by improving the resolution. Second, there could be a learning effect in terms of interactions in VR for participants who had no prior VR experience. In the full study (planned next steps), participants will be given tutorials for immersion in VR. Finally, the verification trials were done with few participants (N=3), limiting our ability to generalize findings. However, we would like to emphasize that our study was designed to identify, create, and implement an experimental setup for testing the ability of immersive VR exercises to elicit genuine responses to different types of stressors. In addition, it aimed to assess the feasibility, tolerability, and acceptability of these exercises through verification trials. This is consistent with previous studies that focused on study development [75]. Because the purpose of the verification trials was to show the feasibility, tolerability, and user acceptability, we collected data from only 3 participants, with the intention to run full trials in the future. It is worth noting that, despite the limited number of participants, we were able to demonstrate the feasibility, tolerability, and user acceptability of the study.

### Conclusions

In conclusion, we identified, designed, and implemented an experimental setup for testing the ability of immersive VR exercises to elicit genuine responses corresponding to different types of stressors and tested its feasibility, tolerability, and acceptability using verification trials. While only a small number of trials were carried out with participants, the verification trials provided the support required to carry out a full study (planned next steps).

A strong point of our study design was the collection of several physiological measures that permitted the collection of quantitative measures complementing that of self-report with the NASA-TLX. With the capture of measures such as HRV and facial expressions, it will be possible to compare how the bWell exercises may physiologically elicit response versus what users perceive to be elicited. Such evaluations may serve to obtain a more complete picture of the in-situ user state and may permit gaining insights on the use of traditional self-report, which has been critiqued as an unreliable index of functional outcomes [76]. In this regard, future work will explore multivariate analysis such as partial least squares [77] and ML techniques such as random forest [78] for predicting individual user responses to different stressors based on multiple input data, including sensor data (eg, HRV and facial expressions) and VR-based behavioral data (eg, response time). Such work will help in identifying and delineating the individual features that confer stress resilience and will guide us in helping individuals reach their full potential.

## Appendix

Multimedia Appendix 1 Description of the timeline for different task and recovery, Simulator Sickness Questionnaire scores for pre– and post–virtual reality session, and subjective ratings of game user experience satisfaction scale-18 questionnaires.

## Abbreviations

ANS: autonomic nervous system
CPT: continuous performance test
ECG: electrocardiogram
EMG: electromyography
GUESS: game user experience satisfaction scale
HRV: heart rate variability
ML: machine learning
NASA-TLX: NASA-Task Load Index
NVIM: Neurovisceral Integration Model
PPG: photoplethysmography
RMSSD: root mean square of successive differences
SSQ: Simulator Sickness Questionnaire
UI: user interface
VR: virtual reality
VTT: Vagal Tank Theory

## Data Availability

The datasets generated and analyzed during this study are not publicly available due to legislation regarding privacy and security. None of the data for the experiments reported here are available, and none of the experiments were preregistered.
