# Supplementary material for: An Integrated Platform Combining Immersive Virtual Reality and Physiological Sensors for Systematic and Individualized Assessment of Stress Response (bWell): Design and Implementation Study
Source: JMIR Form Res. 2025 Mar 4;9:e64492. doi: 10.2196/64492 (PMC11920663; doi:10.2196/64492)
Supplement: Multimedia Appendix 1 [file formative_v9i1e64492_app1.docx]

**Supplementary Material**

**For**

**“An integrated platform combining immersive VR and physiological sensors for systematic and individualized assessment of stress response (bWell): Design and Implementation of an Innovative Experimental Set-up”**

**Table S1.** Description of the timeline for different task and recovery. The initial baseline was 5 minutes long, whereas the consequent VR exercises and recovery were 2 minutes in duration.

| **Exercise** | **Duration (min)** |
| --- | --- |
| Tent (baseline) | 5 |
| Tent (frustration) | 2 |
| Tent (recovery) | 2 |
| Mole (mental/temporal demand) | 2 |
| Tent (recovery) | 2 |
| Stroll (physical demand) | 2 |
| Tent (recovery) | 2 |
| Stroll+CPT (physical + mental demand) | 2 |
| Tent (recovery) | 2 |

**Table S2.** Description of the SSQ scores for pre- and post-VR session. Total SSQ scores based on the subscale ratings were computed based on ^69^. Here, P1, P2 and P3 denote participant 1, participant 2 and participant 3, SSQ = Simulator Sickness Questionnaire and VR = virtual reality.

|  | **P1**  **Pre Post** | **P2**  **Pre Post** | **P3**  **Pre Post** |
| --- | --- | --- | --- |
| General discomfort | 0 1 | 0 0 | 0 1 |
| Fatigue | 1 0 | 2 0 | 0 0 |
| Headache | 0 0 | 0 0 | 0 0 |
| Eye-strain | 0 0 | 1 2 | 1 1 |
| Difficulty focusing | 1 0 | 0 0 | 0 0 |
| Salivation increasing | 0 0 | 0 0 | 0 0 |
| Sweating | 0 0 | 0 0 | 0 0 |
| Nausea | 0 0 | 0 0 | 0 0 |
| Difficulty concentrating | 1 0 | 0 0 | 0 0 |
| Fullness of the head | 0 0 | 0 0 | 0 0 |
| Blurred vision | 0 0 | 0 0 | 0 0 |
| Dizziness (eyes open) | 0 0 | 0 0 | 0 0 |
| Dizziness (eyes closed) | 0 0 | 0 0 | 0 0 |
| Vertigo | 0 0 | 0 0 | 0 0 |
| Stomach awareness | 1 0 | 0 0 | 0 0 |
| Burping | 0 0 | 0 0 | 0 0 |
| **Total SSQ score** | **4 1** | **3 2** | **1 2** |

**Table S3.** Description of the subjective ratings of GUESS-18 questionnaires. The scores of 8 constructs/subscales were used for the computation of overall GUESS score to infer usability and immersiveness of the designed VR exercises. Here, P1, P2 and P3 denote participant 1, participant 2 and participant 3.

| **Constructs** | **Scores/Ratings**  **P1 P2 P3** |
| --- | --- |
| **Usability** U1  U2 | 7 6 6  6 6 6 |
| **Narratives** N1  N2 | 6 - 5  6 - 5 |
| **Play engrossment** PE1  PE2 | 6 6 7  6 6 7 |
| **Enjoyment** E1  E2 | 7 4 6  1 3 5 |
| **Creative freedom**  CF1  CF2 | 6 2 5  6 2 5 |
| **Audio aesthetics** AA1  AA2 | 6 2 6  6 5 6 |
| **Personal gratification** PG1  PG2 | 7 6 7  7 6 7 |
| **Visual aesthetics** VA1  VA2 | 7 6 5  7 6 5 |
| **Overall GUESS score** | **43.17** |
